# Citrus Peel Hydrolates as By-Products of Hydrodistillation: Volatile Characterisation and the Role of Enzymatic Pretreatment

**DOI:** 10.3390/molecules31071118

**Published:** 2026-03-28

**Authors:** Maja Dent, Marija Penić, Antonela Ninčević Grassino, Krunoslav Aladić, Stela Jokić, Igor Jerković

**Affiliations:** 1 Faculty of Food Technology and Biotechnology, University of Zagreb, Pierottijeva 6, 10000 Zagreb, Croatia; doktoricamare@gmail.com (M.P.); aninc@pbf.hr (A.N.G.); 2 Faculty of Food Technology Osijek, University of Josip Juraj Strossmayer in Osijek, Franje Kuhača 18, 31000 Osijek, Croatia; krunoslav.aladic@ptfos.hr (K.A.); sjokic@ptfos.hr (S.J.); 3 Faculty of Chemistry and Technology, University of Split, Ruđera Boškovića 35, 21000 Split, Croatia; 4 Mediterranean Institute for Life Sciences, University of Split, Meštrovićevo šetalište 45, 21000 Split, Croatia

**Keywords:** citrus peel, hydrolates, enzymatic pretreatment, hydrodistillation, HS–SPME GC–MS

## Abstract

This study comprehensively characterised the volatile composition of hydrolates obtained as by-products of the hydrodistillation of orange, mandarin, and clementine peels. Enzymatic pretreatments using pectinase, cellulase, xylanase, or their mixture were applied in purified water or citrate buffer (pH 5) prior to Clevenger hydrodistillation, and volatile profiles were analysed by HS–SPME GC–MS. Across all citrus species, hydrolates were dominated by oxygenated monoterpenes and alcohols, with *α*-terpineol and linalool identified as the principal constituents. Statistical analysis suggested differences in hydrolate volatile composition following enzymatic pretreatment in citrate buffer with cellulase, xylanase, or their combination (*p* < 0.05); notably, *α*-terpineol content in mandarin hydrolates nearly doubled after these treatments. Enzyme-free reflux extraction in water also led to observable changes in volatile profiles (*p* < 0.05), highlighting the importance of including both untreated and enzyme-free controls when evaluating enzymatic effects. The study also illustrates the distinct distribution of dominant volatiles between hydrolates with prevailing *α*-terpineol. These findings demonstrate the potential of enzymatic hydrodistillation for the valorisation of citrus peel by-products by enabling modulation of hydrolate volatile composition and supporting more sustainable use of citrus processing residues.

## 1. Introduction

Citrus peels are a major by-product of global citrus production and are widely recognized as a rich source of bioactive compounds. Despite their potential, much of this biomass remains underutilized, with large quantities discarded or incinerated, leading to both environmental and economic losses. Essential oils extracted from citrus peels have been extensively studied and are highly valued in the food, fragrance, and pharmaceutical industries [1,2,3,4,5]. However, the hydrodistillation process used for their isolation also generates substantial by-products—most notably hydrolates [6,7,8] and water extracts [9]—which remain comparatively understudied, despite containing dissolved volatile and bioactive compounds. Hydrolates, also known as hydrosols, are aromatic waters produced as co-products during the hydrodistillation of plant material. They consist of distillation water enriched with trace amounts of volatile constituents and microdroplets of essential oils [10,11]. Although long overshadowed by essential oils, hydrolates are increasingly recognised for their potential. They have already found application in the food and cosmetics industries, where they are valued for their mild fragrance and gentle bioactive properties. Building on these attributes, hydrolates—like essential oils—show strong potential for a wide range of industrial applications, offering further opportunities to valorise citrus peel biomass and promote more sustainable practices across multiple sectors [8,10,11,12]. A key characteristic supporting their wider application—especially in food-related uses—is that hydrolates are considered safe for human consumption, similar to essential oils, which are listed as Generally Recognised as Safe (GRAS) in the U.S. Code of Federal Regulations [13]. Only a few studies have characterized citrus hydrolates, identifying *α*-terpineol, linalool, and geraniol as the main constituents in hydrolates from orange, mandarin, lime, pomelo, lemon, and Buddha’s hand citron peels [6,7,8], as well as in bitter orange flowers [14,15,16,17]. To date, research on citrus hydrolates has primarily focused on products obtained from orange and mandarin peels and bitter orange flowers by steam distillation, as well as from Buddha’s hand citron peels and bitter orange flowers by hydrodistillation [6,7,8,14,15,16,17]. In contrast, hydrolates derived from other citrus peels, particularly clementine, remain largely unexplored, highlighting a significant knowledge gap, especially regarding hydrodistillation and its potential combination with enzymatic pretreatments to improve hydrolate quality. This gap is further underscored by the low essential oil yields typically obtained from citrus peels by Clevenger hydrodistillation, which has prompted the exploration of various pretreatment strategies—such as ultrasound, enzymatic treatment, and salt-assisted extraction—to enhance both yield and product quality [18,19,20,21]. Among these approaches, enzymatic pretreatment is particularly promising, as enzymes such as cellulase, pectinase, xylanase, and hemicellulase can degrade plant cell wall components, thereby facilitating the release of soluble volatile compounds and influencing the composition of both essential oils and hydrolates [18,19,21]. However, existing studies remain limited and often lack appropriate experimental controls, making it difficult to clearly distinguish the effects of individual enzymes, enzyme mixtures, and reaction conditions. The frequent use of only untreated controls [19,21] or pretreatments without enzymes [18] further hampers reliable assessment of enzymatic contributions to the final composition of essential oils and hydrolates.

In this context, the aim of the present study is to comprehensively evaluate the volatile composition of hydrolates obtained as by-products of hydrodistillation during the isolation of essential oils from orange, mandarin, and clementine peels. Enzymatic pretreatments were applied to peels before hydrodistillation to assess their impact on hydrolate quality. The treatments included individual enzymes—pectinase, cellulase, and xylanase—as well as a mixture of these enzymes, applied in purified water and citrate buffer (pH 5). Two control treatments were also performed: hydrodistillation without pretreatment and hydrodistillation with reflux pretreatment in water or buffer without enzyme addition, allowing assessment of the specific contribution of the enzymes. Volatile profiles of hydrolates were characterised using HS–SPME GC–MS, and statistical analyses were conducted to determine the effect of enzymatic pretreatment on the distribution and amount of volatile compounds in hydrolates from orange, mandarin, and clementine peels.

## 2. Results and Discussion

### 2.1. Volatile Composition of Orange, Mandarin and Clementine Peel Hydrolates

**Figure 1:**
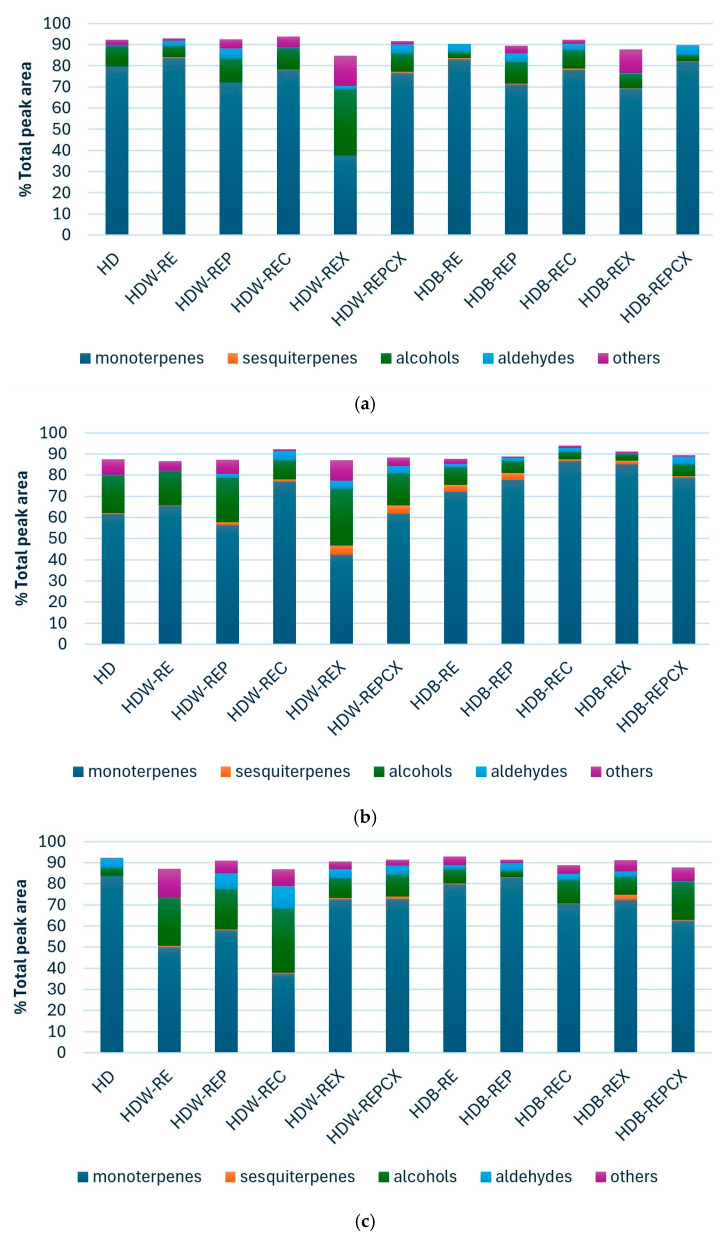
Groups of volatile compounds in (**a**) orange, (**b**) mandarin and (**c**) clementine peel hydrolate determined by HS–SPME GC–MS analysis. HD—hydrodistillation without pretreatment (no-pretreatment control); HDW—hydrodistillation with water (no-enzyme control); HDB—hydrodistillation with buffer (no-enzyme control); RE—reflux extraction without enzyme; REP—reflux extraction with pretreatment assisted with enzyme pectinase; REC—reflux extraction with pretreatment assisted with enzyme cellulase; REX—reflux extraction with pretreatment assisted with enzyme xylanase; REPCX—reflux extraction with pretreatment assisted with mixture of enzymes (pectinase/cellulose/xylanase).

The volatile composition of citrus peel hydrolates has been only sparsely investigated. Existing studies have identified *α*-terpineol, linalool, and geraniol as the main constituents of hydrolates obtained from the peels of orange, mandarin, lime, pomelo, lemon, and Buddha’s hand citron [6,7,8], as well as from bitter orange flowers [14,15,16,17] (Table 1). To date, research has focused primarily on hydrolates produced by steam distillation of orange, mandarin, and bitter orange peels and flowers, and by hydrodistillation of Buddha’s hand citron peels and flowers. In contrast, hydrolates derived from the peels of other citrus fruits, including clementine, remain largely unexplored. This highlights a significant knowledge gap, particularly regarding hydrodistillation processes and their potential combination with enzymatic pretreatment to improve hydrolate quality. In this study, hydrolates obtained as by-products of Clevenger hydrodistillation during essential oil isolation from enzymatically pretreated orange, mandarin, and clementine peels were characterised by HS–SPME GC–MS (Tables S1–S3, Supplementary Material). To compare volatile profiles across pretreatments, a heat map with hierarchical clustering was generated based on HS–SPME GC–MS data (Figure 1a–c). Overall, the hydrolates of all three citrus species were dominated by monoterpenes (83.74%, 86.74%, and 83.84% for orange, mandarin, and clementine, respectively) and alcohols (30.98%, 26.94%, and 30.52%), indicating a consistent volatile composition among the studied citrus peel hydrolates.

### 2.2. Comparison of the Volatile Composition of Hydrolates and Essential Oils from Orange, Mandarin, and Clementine Peel

**Figure 2:**
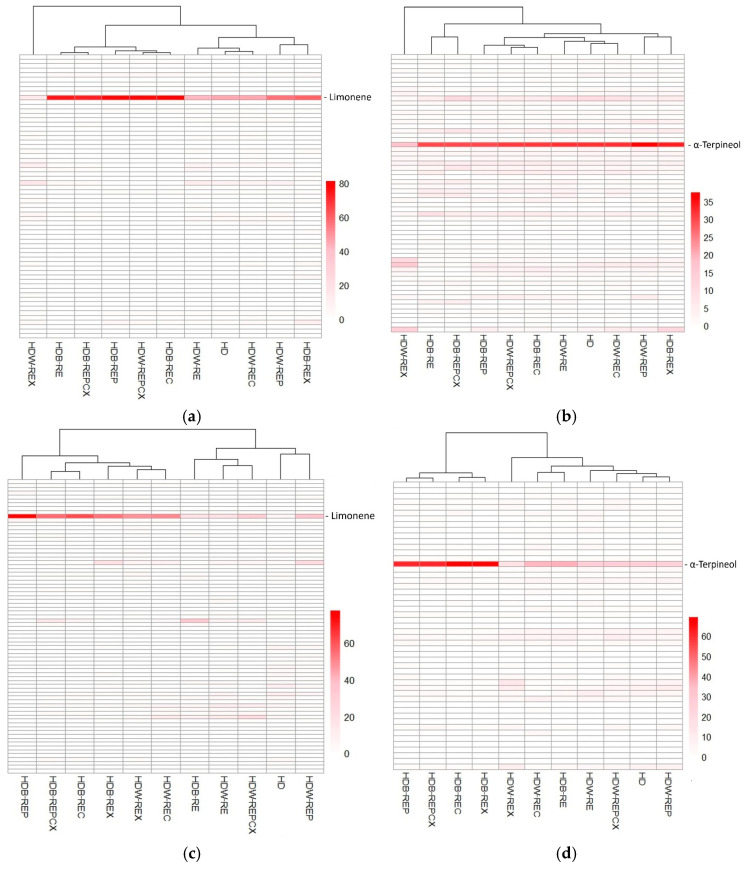

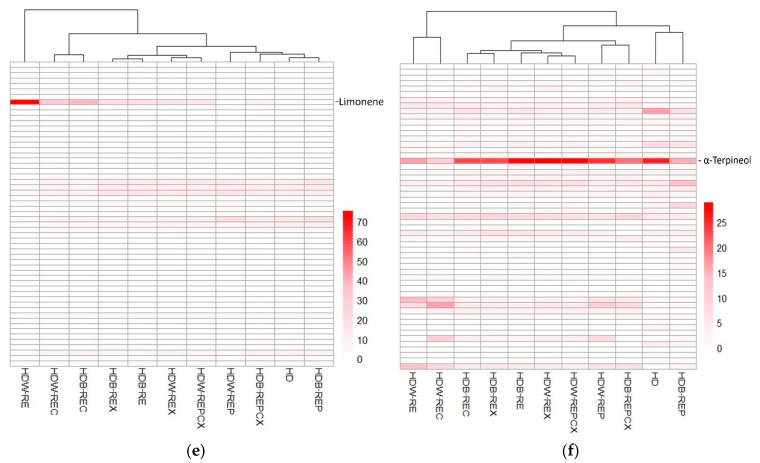
Clustered heatmap of orange, mandarin, and clementine peel compound properties across different pretreatments. The heatmap shows the variation in dominant compound properties ((**a**)—orange, (**c**)—mandarin, (**e**)—clementine–essential oil [22] and (**b**)—orange, (**d**)—mandarin, (**f**)—clementine–hydrolate content) between different pretreatments indicated in the columns. The values are represented by a colour gradient, where lighter colours indicate lower values and red indicates higher values. Hierarchical clustering was applied to the columns, revealing similarities in pretreatment responses. The most dominant compound is indicated in red. HD—hydrodistillation without pretreatment (no-pretreatment control); HDW—hydrodistillation with water (no-enzyme control); HDB—hydrodistillation with buffer (no-enzyme control); RE—reflux extraction without enzyme; REP—reflux extraction with pretreatment assisted with enzyme pectinase; REC—reflux extraction with pretreatment assisted with enzyme cellulase; REX—reflux extraction with pretreatment assisted with enzyme xylanase; REPCX—reflux extraction with pretreatment assisted with mixture of enzymes (pectinase/cellulose/xylanase).

The volatile composition of orange, mandarin, and clementine peel hydrolates was compared with that of the corresponding essential oils, the quantities of which were obtained in the previous paper by Penić et al. [22]. As expected, hydrolates were dominated by more hydrophilic compounds than essential oils, reflecting the dissolution of water-soluble volatile organic compounds in the aqueous phase [11]. Accordingly, the main volatile constituents of hydrolates differed substantially from those of essential oils. Hydrolates were characterised by a predominance of oxygenated monoterpenes and alcohols (Figure 1a–c), with *α*-terpineol and linalool as the major compounds (Tables S1–S3, Supplementary Material). *α*-Terpineol was the most abundant constituent, accounting for up to 37.52%, 69.14%, and 28.86% of the total peak area in orange, mandarin, and clementine peel hydrolates, respectively. These values are markedly higher than those reported for the corresponding essential oils (up to 0.78%, 23.61%, and 2.14%) [22]. The elevated *α*-terpineol levels in hydrolates compared to citrus peels reported elsewhere (orange 4.41%, mandarin 10.1%) are attributed to hydrodistillation used in this study, as opposed to steam distillation applied in other works [7,8]. Cluster analysis based on HS–SPME GC–MS data further confirmed distinct compositional patterns between essential oils and hydrolates (Figure 2a–f). Essential oils of all three citrus peels were dominated by monoterpene hydrocarbons, particularly limonene (up to 81.16%, 77.50%, and 75.29%) [22], whereas limonene was present in hydrolates only at low levels (up to 2.21%, 0.73%, and 2.30%) (Tables S1–S3, Supplementary Material). These findings are consistent with previous reports showing substantially lower limonene contents in citrus peel hydrolates compared to essential oils [6,7,8], while essential oils consistently contain high limonene proportions [9,18,19,23,24,25]. In addition, orange and clementine peel hydrolates contained higher proportions of linalool (up to 11.15% and 16.45%) than their essential oils (up to 1.70% and 2.57%), while mandarin peel hydrolate showed a linalool content comparable to its essential oil [22]. Higher linalool levels in orange and mandarin peel hydrolates have also been reported previously [7]. By contrast, bitter orange flower hydrolates are considerably richer in linalool (15.4–56.5%), underscoring pronounced compositional differences between flower and peel hydrolates [14,15,16,17]. Furthermore, hydrolates exhibited significantly higher total alcohol contents (up to 30.98%, 26.94%, and 30.98%) than essential oils (up to 0.49%, 1.71%, and 1.05%). The dominant alcohols were 3-methylbut-3-en-1-ol and 2-methylbut-3-en-2-ol, which were absent from essential oils and are rarely reported or occur at much lower levels in citrus peel hydrolates [6,7,8]. Ketones such as propan-2-one (up to 14.36%) were detected exclusively in hydrolates. Conversely, several sesquiterpenes abundant in mandarin peel essential oil (e.g., *t*-cadinol, *δ*-cadinene, and (*E*,*E*)-*α*-farnesene) were absent or detected only in trace amounts in the corresponding hydrolates.

### 2.3. Impact of Enzymatic Pre-Treatments on the Volatile Composition of Orange, Mandarin and Clementine Peel Hydrolates

The influence of enzymatic pretreatment of citrus peels prior to hydrodistillation on the volatile composition of hydrolates was evaluated using Kruskal–Wallis analysis (Table 2); however, the results should be interpreted with caution, as volatile compounds represent components of a compositional profile and may not fully satisfy the assumption of independence. Therefore, the analysis is considered primarily exploratory and descriptive rather than strictly inferential. Orange, mandarin, and clementine peels were pretreated with or without enzymes in water or citrate buffer immediately before hydrodistillation. Significant differences (*p* < 0.05) in hydrolate volatile composition were detected in the distribution of relative abundances of volatile compounds, compared to the untreated control (HD), for reflux extraction in water without enzymes (HDW–RE) and for enzymatic pretreatments with cellulase (HDB–REC), xylanase (HDB–REX), and enzyme mixtures (HDB–REPCX) performed in citrate buffer across all three citrus peels. In contrast, enzymatic pretreatments carried out in water, as well as pretreatments in citrate buffer without enzymes or with pectinase, did not significantly (*p* > 0.05) modify the overall distribution of volatile compounds, which remained comparable to that of the control. Due to the limited number of studies addressing the effect of enzymatic pretreatment on hydrolate composition, these results were compared with the available literature on essential oils. Previous studies have reported a pronounced influence of enzymatic pretreatment on the volatile composition of citrus essential oils [18,19,22]. Importantly, only Penić et al. [22] included both a control without pretreatment and a pretreatment control without enzyme addition, which is necessary for reliable assessment of enzymatic effects.

To better understand the observed differences, it is important to consider the mechanisms by which enzymatic pretreatment affects the plant matrix. Cellulase hydrolyzes *β*-1,4-glycosidic bonds in cellulose, one of the main components of the plant cell wall, reducing crystallinity and increasing matrix porosity [26,27], while xylanase cleaves *β*-1,4-xylosidic bonds in xylan, a hemicellulosic component connecting cellulose and lignin, thereby weakening structural interactions within the cell wall [28]. Pectinase, on the other hand, hydrolyzes *α*-1,4-linked galacturonic acid residues in pectin, leading to the degradation of plant tissues. These enzymatic actions disrupt the overall structure of the cell wall, increase its permeability, and facilitate mass transfer during hydrodistillation, allowing volatile compounds trapped in oil glands or associated with the matrix to be released more readily [29]. Enzyme loading was determined based on preliminary optimization experiments to achieve sufficient hydrolytic activity without enzyme denaturation or substrate inhibition. Hydrolysis was carried out at 50 °C under reflux, with pH and temperature monitored to maintain optimal conditions for each enzyme. Control experiments without enzymes were also performed to distinguish the effects of enzymatic hydrolysis from those of thermal treatment alone. Volatile compounds in citrus peel are retained through physical entrapment within oil glands, adsorption to cell wall polymers, or glycosidic linkage to polysaccharide precursors. Cellulase primarily releases physically trapped compounds, xylanase increases accessibility by loosening the hemicellulosic network, and pectinase promotes the rupture of oil glands by degrading pectic substances [27,28]. The effectiveness of enzymatic hydrolysis is reflected in the accumulation of soluble or reducing sugars in the hydrolysates, which were quantified using the 3,5-dinitrosalicylic acid (DNSA) assay. Cellulase, xylanase, and pectinase were incubated with their respective substrates at 50 °C for 120 min in purified water and citrate buffer (pH 5), and the enzyme activity results were reported in a previous study [22]. Active hydrolysis was observed in purified water (pectinase 50.4 U/mL, cellulase 12.7 U/mL, xylanase 22.3 U/mL) and in citrate buffer (pectinase 41.5 U/mL, cellulase 25.3 U/mL, xylanase 13.5 U/mL). This accumulation of monosaccharides reflects enzymatic activity and the extent of cell wall degradation, and can enhanced release of volatile compounds during hydrodistillation [29].

**Figure 3:**
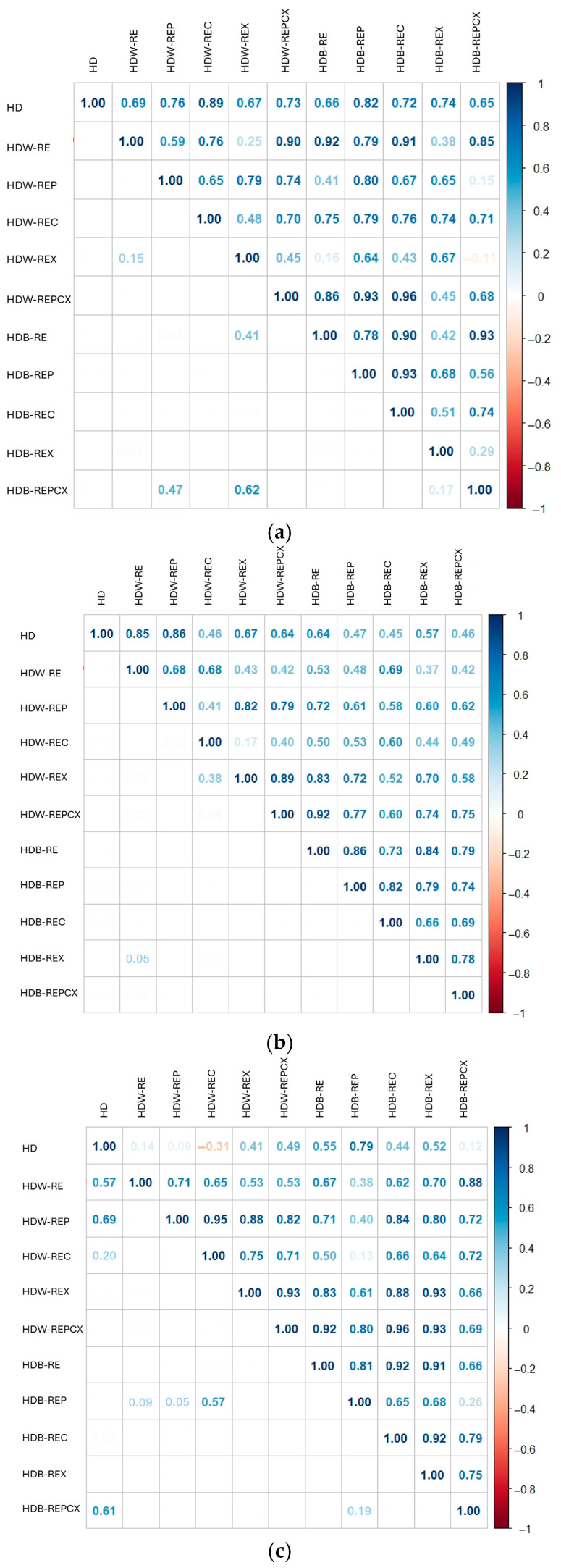
Spearman correlation matrix for the different pretreatments on the hydrolate composition of selected variables of each citrus fruit: (**a**) orange; (**b**) mandarin; (**c**) clementine peel. The upper triangle shows the correlation coefficients, and the lower triangle shows the corresponding *p*-values. Each correlation coefficient quantifies the strength and direction of the monotonic relationship between two variables and ranges from −1 (perfect negative correlation) to 1 (perfect positive correlation). Only statistically significant differences are considered, with the *p*-values indicating a low correlation between the observed variables. In the upper triangle, higher absolute values indicate stronger correlations. Effect of different hydrodistillation pretreatments on the yield of orange, mandarin, and clementine hydrolate. HD—hydrodistillation without pretreatment (no-pretreatment control); HDW—hydrodistillation with water (no-enzyme control); HDB—hydrodistillation with buffer (no-enzyme control); RE—reflux extraction without enzyme; REP—reflux extraction with pretreatment assisted with enzyme pectinase; REC—reflux extraction with pretreatment assisted with enzyme cellulase; REX—reflux extraction with pretreatment assisted with enzyme xylanase; REPCX—reflux extraction with pretreatment assisted with mixture of enzymes (pectinase/cellulose/xylanase).

Overall, the results indicate that enzymatic pretreatment with selected enzymes—cellulase, xylanase, and the enzyme mixture—performed in citrate buffer was associated with statistically detectable shifts in the volatile composition of orange, mandarin, and clementine peel hydrolates, whereas enzymatic pretreatment in water showed no statistically detectable differences relative to the control. These observations should be interpreted as relative compositional shifts rather than definitive improvements in hydrolate quality, considering the semi-quantitative nature of HS-SPME GC–MS analysis. This behaviour contrasts with that observed for essential oils, for which the same enzymatic pretreatments markedly affected oil quality [22]. These differences are clearly illustrated in the heat map (Figure 1a–c), which shows a predominance of monoterpenes following enzymatic pretreatment in buffer, while pretreatment in water results in higher proportions of alcohols. These patterns therefore reflect relative changes in the proportions of compound groups rather than absolute quantitative differences. The specific effects of enzymatic pretreatment on the volatile composition of hydrolates are discussed in detail in the following sections for each type of citrus peel. In addition, Spearman’s rank correlation analysis was used to evaluate the similarity of volatile profiles among hydrolates obtained after different pretreatments. The analysis revealed positive correlations between the samples and a high overall compositional similarity with the untreated control (HD). Although the Kruskal–Wallis test indicated statistically significant differences in the distribution of compound abundances, the high Spearman correlation coefficients (*r* = 0.89; 0.86; 0.79) suggest that the overall structure of the volatile profiles remained largely preserved, with dominant compounds maintaining similar relative rankings across the samples. In this context, enzymatic pretreatments induce moderate quantitative shifts in specific constituents, while the general pattern of the volatile profile remains comparable to that of the control (Figure 3a–c).

#### 2.3.1. Orange Peel

In orange peel hydrolates, the main oxygenated monoterpenes identified were *α*-terpineol (17.55–37.52%), linalool (0.61–11.15%), 4-terpineol (1.02–8.60%), and *p*-mentha-1,8(10)-dien-9-ol (1.26–8.55%), followed by the alcohols 3-methylbut-3-en-1-ol (0.50–15.32%) and 2-methylbut-3-en-2-ol (0.37–11.21%). A notable proportion of propan-2-one (0.05–14.36%) was also detected. Overall, the volatile composition of orange peel hydrolates is consistent with previous reports [7,8], although the relative proportions of dominant compounds differ. For example, earlier studies [7,8] reported higher linalool (34.88%) and lower *α*-terpineol (4.41%) compared to our findings, likely due to differences in hydrolate isolation methods. Steam distillation generally yields higher linalool, while hydrodistillation favours *α*-terpineol. This is consistent with hydrolates from bitter orange flowers, where hydrodistillation produced 16.58–17.5% linalool and 20.7–23.7% *α*-terpineol [14,15,16,17]. Enzymatic pretreatment of orange peel in citrate buffer prior to hydrodistillation influenced the volatile profile of the hydrolate. The highest linalool content (up to 11.15%) was observed after pretreatment with the enzyme mixture in buffer (HDB–REPCX). Kruskal–Wallis analysis indicated differences in the overall volatile composition among enzymatic pretreatments with cellulase, xylanase, and the enzyme mixture in buffer (*p* < 0.05, Table 2); however, their interpretation is limited by the compositional nature of the data. However, these treatments did not markedly increase the proportion of the dominant compound *α*-terpineol. The highest *α*-terpineol levels were 34.42% with xylanase in buffer (HDB–REX) and 37.52% with pectinase in water (HDW–REP), compared to 32.49% in the untreated control (HD) and 32.73% in the control without enzyme addition (HDB–RE). Spearman’s rank correlation further supported these observations, showing positive correlations for orange peel pretreatments with cellulase (HDB–REC, *r* = 0.72), xylanase (HDB–REX, *r* = 0.74), and the enzyme mixture (HDB–REPCX, *r* = 0.65) relative to the untreated control (Figure 3a). These results indicate that enzymatic pretreatment in buffer modifies the volatile composition of orange peel hydrolates, particularly for minor compounds, but does not strongly affect the major compound *α*-terpineol.

#### 2.3.2. Mandarin Peel

Mandarin peel hydrolates were dominated by oxygenated monoterpenes, particularly *α*-terpineol (16.27–69.14%), followed by *trans*-carveol (1.63–5.95%), *p*-mentha-1,8(10)-dien-9-ol (1.13–5.50%), and perillyl alcohol (1.31–8.03%). Significant amounts of 3-methylbut-3-en-1-ol (0.82–9.65%), 2-methylbut-3-en-2-ol (0.46–12.20%), and propan-2-one (0.53–9.66%) were also detected. Linalool content was low (0.13–2.44%) regardless of pretreatment. Enzymatic pretreatment of mandarin peel in buffer had a pronounced effect on *α*-terpineol, doubling its proportion to 60.94–69.14%, whereas pretreatment in water increased it only to 16.27–35.51%. The untreated control (HD) contained 27.88% *α*-terpineol. Differences in volatile composition compared to previous reports [7], which found 10.1% *α*-terpineol and 17.5% linalool, are likely due to differences in isolation methods, as the previous study used steam distillation, while hydrodistillation was applied here. High levels of *α*-terpineol have also been reported in other citrus peel species, including Buddha’s hand citron after hydrodistillation (44.7%) and lemon after steam distillation (29.98%) [6,8]. Spearman’s correlation analysis showed positive correlations for the buffer pretreatments: HDB–REC (*r* = 0.45), HDB–REX (*r* = 0.57), and HDB–REPCX (*r* = 0.46), as well as a strong positive correlation for the enzyme-free water control (HDW–RE, *r* = 0.85), highlighting the clear impact of enzymatic pretreatment on the volatile composition (Figure 3b). These results are consistent with the Kruskal–Wallis analysis, which indicated differences in the volatile profile of citrus peel hydrolates among enzymatic pretreatments in citrate buffer and the enzyme-free water control (*p* < 0.05, Table 2); however, these findings are best considered exploratory.

#### 2.3.3. Clementine Peel

In clementine peel hydrolates, oxygenated monoterpenes predominated, including linalool (1.20–16.45%), *α*-terpineol (11.09–28.86%), *trans*-carveol (2.56–13.63%), carvone (0.22–8.00%), and *cis*-isopiperitenone (0.65–4.11%). Alcohols such as 3-methylbut-3-en-1-ol (0.62–16.75%) and 2-methylbut-3-en-2-ol (0.29–14.02%), the aldehyde furfural (0.01–10.73%), and propan-2-one (0.02–13.68%) were also detected. This composition is consistent with reports on hydrolates from peels of other citrus fruits, including orange, mandarin, lime, lemon, citron, and Buddha’s hand citron [6,7,8]. As data on clementine peel hydrolates are limited, comparisons are made with these related citrus species. Enzymatic pretreatment of clementine peel did not result in a substantial increase in the main volatiles, *α*-terpineol and linalool. The highest linalool content (16.45%) was observed in the hydrolate without pretreatment (HD), while *α*-terpineol levels showed slight increases after treatment with enzyme-free buffer (HDB–RE, 28.21%) and after enzyme pretreatments in water with xylanase (HDW–REX, 28.86%) or a xylanase mixture (HDW–REPCX, 28.39%), compared to the untreated control (HD, 25.92%). Spearman’s correlation analysis revealed positive relationships between the untreated control and both enzyme-free and enzymatic pretreatments (HDB–REC, *r* = 0.44; HDB–REX, *r* = 0.52; HDW–REX, *r* = 0.41; HDW–REPCX, *r* = 0.49) (Figure 3c). The Kruskal–Wallis test indicated differences in the overall volatile composition of citrus peel hydrolates among enzymatic pretreatments in citrate buffer (*p* < 0.05, Table 2), whereas no differences were observed for treatments in water. These results indicate that, although positive Spearman correlations were observed, the impact of enzymatic pretreatment on specific compounds such as *α*-terpineol does not always correspond with its overall effect on the volatile profile.

Due to the limited literature on hydrolates obtained from orange, mandarin, and clementine peels, further research on these by-products is necessary. The results of this study indicate that enzymatic pretreatment is a suitable method for hydrodistillation, as it influences the composition of volatile compounds in orange, mandarin, and clementine peel hydrolates. Statistical analysis of HS–SPME GC–MS data (Table 2, Figure 3a–c) confirmed that enzymatic pretreatment in buffer significantly affects the dominant volatile compounds in orange, mandarin, and clementine hydrolates (*p* < 0.05), particularly when cellulase, xylanase, or their mixture is used. In contrast, enzymatic pretreatment in water did not lead to significant changes in hydrolate volatile composition. However, pretreatment by reflux extraction in water without enzymes was also significant (*p* < 0.05), indicating that soaking the peel alone can release volatile compounds and alter the final hydrolate profile. Therefore, to accurately assess the effectiveness of enzymatic pretreatment, it is essential to include both control samples: one without pretreatment and one without enzymes under identical extraction conditions. As shown by the results of this study, both controls are necessary for a reliable evaluation of the specific contribution of enzymes to changes in the volatile profile. Given the scarcity of studies on hydrolates and hydrodistillation by-products, further research comparing different pretreatment approaches is required to identify the most effective methods for improving the volatile composition of hydrolates.

## 3. Materials and Methods

### 3.1. Chemicals

The following chemicals were used: citrate acid (Gram-Mol, Zagreb, EU, Croatia), sodium hydroxide (Lach-ner, Brno, Czech Republic), cellulase (from *Aspergillus niger*) (Sigma-Aldrich, Tokyo, Japan), pectinase (from *Aspergillus niger*) (Sigma-Aldrich, Buchs, Switzerland), xylanase (from Theryomyces, expressed in *Aspergillus oryzae*) (Sigma-Aldrich, Søborg, Denmark), C_9_–C_25_ alkanes (Eurisotop, Saint-Aubin, France).

### 3.2. Extraction Procedure

The collection of citrus peels—orange (*Citrus sinensis*), mandarin (*Citrus reticulata*), and clementine (*Citrus clementine*)—the preparation of citrus peel for enzymatic pretreatment, as well as Clevenger hydrodistillation are described in detail in a previous paper [22]. Briefly, enzymatic pretreatment included as a follow: (i) reflux extraction with enzymes (pectinase, REP; cellulase, REC; xylanase, REX; pectinase/cellulase/xylanase, REPCX) in purified water or citrate buffer (pH 5), and control pretreatments: (ii) reflux extraction without enzymes (RE) in purified water (HDW) or citrate buffer (pH 5) (HDB) as control samples. A control determination without prior pretreatment (HD) was also conducted. The enzyme activities (cellulase, pectinase, and xylanase) in purified water and citrate buffer (pH 5) were confirmed by the colorimetric 3,5-Dinitrosalicylic Acid (DNSA) method in a previously published work [22]. The hydrolate was separated one hour after the end of Clevenger hydrodistillation, once the apparatus had cooled. The hydrolate samples were stored at 4 °C until analysis.

### 3.3. Headspace Solid-Phase Microextraction (HS–SPME) with Gas Chromatography-Mass Spectrometry (GC–MS) Analysis

HS–SPME was performed automatically with the PAL Auto Sampler System (PAL RSI 85, CTC Analytics AG, Schlieren, Switzerland) using the fiber covered with a layer of carbon wide range/polydimethylsiloxane (Carbon WR/PDMS) (Agilent Technologies, Palo Alto, Santa Clara, CA, USA). The fiber was conditioned prior to extraction according to Agilent Technologies’ instructions. The hydrolate (1 mL) was placed in a 15 mL glass vial and hermetically sealed. The vial was maintained in a water bath at 60 °C during equilibration (15 min) and HS–SPME (45 min) under constant stirring (1000 rpm) with a magnetic stirrer. After the sampling, the fiber was withdrawn into the needle and inserted into the GC injector (250 °C) for 6 min, where the extracted volatiles were thermally desorbed directly to the GC column. GC–MS was performed on an Agilent Technologies 8890A gas chromatograph (Palo Alto, CA, USA) coupled to 5977E mass detector (Agilent Technologies, Santa Clara, CA, USA). The compounds were analysed on a HP–5MS column (Agilent Technologies, Santa Clara, CA, USA) 30 m × 0.25 mm with a stationary phase (5% diphenyl/95% dimethylpolysiloxane) and a film thickness of 0.25 μm. The GC operating conditions were: 250 °C injector temperature; 300 °C detector temperature; column temperature: 2 min isothermal at 70 °C, followed by a temperature gradient of 3 °C/min from 70 °C to 200 °C and further retention for 15 min at constant temperature. The carrier gas was helium with a flow rate of 1.0 mL/min; the MSD (EI mode) was operated at 70 eV; the mass range was set from 30 to 300 amu. The compounds were identified by comparing their retention indices (RIs), based on the retention times of C_9_–C_25_ alkanes, with those in the literature (National Institute of Standards and Technology) [30] and their mass spectra with those from the Wiley 9 (Wiley, New York, NY, USA) and NIST Chemistry WebBook (2023) [31] mass spectral libraries. Percent composition was determined using the normalisation method (without correction factors). The HS–SPME/GC–MS was performed in duplicate, and the results obtained were expressed as the average percentage of the peak areas.

### 3.4. Statistical Analysis

The normality of the data was assessed using the Shapiro–Wilk test, which indicated a significant deviation from normality. Accordingly, the Kruskal–Wallis test was used to evaluate whether pretreatments prior to Clevenger hydrodistillation differed in volatile composition across citrus types. Due to the absence of replicate measurements for individual volatile compounds, statistical comparisons at the level of single compounds were not possible. Therefore, the Kruskal–Wallis test was applied to the complete set of relative abundances of identified compounds to explore differences in the overall distribution of volatile profiles among citrus species. Given the compositional nature of the data and the potential lack of independence among compounds, the results of this analysis were interpreted primarily in an exploratory and descriptive context rather than as strictly inferential. A cluster heatmap analysis was performed to visualise variation in volatile compound properties of orange, mandarin, and clementine hydrolates across the different pretreatments. The heatmap was generated using the pheatmap package (Version 1.0.12) in R, with hierarchical clustering applied to the columns to identify similarity patterns and grouping trends [32]. Euclidean distance was used as the distance metric, complete linkage as the clustering method, and no data scaling was applied to the values. Spearman’s rank correlation analysis was used to evaluate the similarity of volatile profiles among hydrolates obtained after different pretreatments. This non-parametric approach was selected because HS–SPME GC–MS provides semi-quantitative data expressed as relative abundances of compounds, often including non-detected values and highly skewed distributions. Spearman correlation therefore allows comparison of samples based on the relative ranking and dominance pattern of volatile constituents rather than absolute quantitative differences Spearman rank correlations were calculated, and *p*-values were adjusted for multiple testing using the Benjamini–Hochberg false discovery rate (FDR) correction. All statistical analyses were conducted using R (R version 4.3.1) in RStudio version 1.0.12, with statistical significance set at *p* < 0.05.

## 4. Conclusions

This study offers a comprehensive characterisation of the volatile composition of hydrolates obtained as by-products of the hydrodistillation of orange, mandarin, and clementine peels, thereby addressing a notable gap in the literature, particularly regarding clementine peel hydrolates. In all three citrus species, the hydrolates were consistently dominated by oxygenated monoterpenes and alcohols, which formed the main classes of volatile compounds. *α*-terpineol and linalool were identified as the principal constituents, with *α*-terpineol especially abundant in mandarin peel hydrolates, where its content increased markedly following specific pretreatments. The results show that enzymatic pretreatment is a viable strategy for modulating the volatile composition of citrus peel hydrolates during hydrodistillation. Statistical analysis using the Kruskal–Wallis test indicated differences in the volatile profiles of all three citrus species after enzymatic pretreatment in citrate buffer (pH 5) (*p* < 0.05), especially with cellulase, xylanase, or their combination. Enzymatic pretreatment in water did not show notable changes in hydrolate composition. Reflux extraction in water without enzymes also led to differences in the volatile profile (*p* < 0.05), suggesting that soaking the peel alone can facilitate the release of volatile compounds. This finding highlights the importance of including both an untreated control and a control without enzyme addition under identical extraction conditions to accurately assess the specific contribution of enzymatic pretreatment. Given the limited number of studies on citrus hydrolates and other hydrodistillation by-products, further research is warranted. Future investigations should systematically compare different pretreatment strategies to identify the most effective approaches for enhancing the volatile composition and overall value of these underexplored by-products.

## Figures and Tables

**Table 1:** A survey of studies on citrus hydrolate composition detailing the tested plant matrix, isolation procedure and processing parameters, and identified major compounds.

Plant Scientific Name	Methodology	Major Compounds	Reference
*Citrus aurantium*	Extraction procedure: steam distillation; Parameters: 15 g dry pollens, 210 mL water, 1 h distillation; (ultrasound–microwave); Parameters: 15 g dry pollens, 210 mL water, processing conditions (ultrasound: 7 min, 90 W; microwave: 75 s, 280 W)	Flower bud: linalool (56.5%), *α*-terpineol (13.0%), *trans*-geraniol (7.9%)	[14]
*Citrus medica* var. *sarcodactylus*	Extraction procedure: hydrodistillation (Clevenger–type apparatus); Parameters: 16 g fresh exocarp, (ratio plant/water not mentioned), 3 h distillation	Fruit exocarp: *α*-terpineol (44.7%), terpinen-4-ol (21.6%), *α*-citral (8.0%), *cis*-geraniol (7.2%), *β*-citral (5.8%)	[6]
*Citrus aurantium*	Extraction procedure: steam distillation (Clevenger–type apparatus); Parameters: 100 g dry flowers, (ratio plant/water not mentioned), 4 h distillation	Flower: linalool (16.58%), neryl acetate (6.48%), nerolidol (5.87%), linalyl acetate (5.0%), limonene (4.79%)	[15]
*Citrus sinensis*, *Citrus reticulata*, *Citrus maxima* and *Citrus aurantifolia*	Extraction procedure: steam distillation (Clevenger–type apparatus); Parameters: 100 g dry peel, 1.5 L water, 1.5 h distillation	Peel: *C. sinensis*: linalool (34.8%); *C. reticulata*: linalool (17.5%), *α*-terpineol (10.1%), *trans*-carveol (12.2%), citronellol (16.4%); *C. maxima*: *trans*-linalooloxide (21.3%), *α*-terpineol (13.0%), *cis*-linalool oxide (furanoid) (10.3%); *C. aurantifolia*: geranial (18.3%), nerol (15.8%), neral (15.3%), geraniol (13.1%), *α*-terpineol (14.6%)	[7]
*Citrus aurantium*	Extraction procedure: steam distillation and hydrodistillation of fresh flowers (ratio plant/water and time of distillation not mentioned)	Flower: linalool (44.1%), *α*-terpineol (23.7%), methyl anthranylate (4.2%)	[16]
*Citrus sinensis*, *Citrus limon*, *Citrus medica*	Extraction procedure: steam distillation; Parameters: 100 g fresh peel, 300 mL water (isolation time not mentioned),	Peel: *C. limon*: geraniol (48.27%), *α*-terpineol (29.98%); *C. sinensis*: terpinolene (12.41%), *α*-terpineol (4.41%); *C. medica*: citral (17.4%), *α*-terpineol (16.81%)	[8]
*Citrus aurantium*	Extraction procedure: hydrodistillation (Clevenger–type apparatus); Parameters: 100 g dry flowers, (ratio plant/water not mentioned), 3.5 h distillation	Flower: laboratory obtained samples: geraniol (26.6%), *α*-terpineol (20.7%), linalool (15.4%), benzene acetaldehyde (5.5%); traditional samples: linalool (44.1%), methyl anthranilate (11.8%), *cis*-linalool oxide (6.1%); industrial samples: 1,8-cineol (15.9%), linalool (13.8%), α-terpineol (6.6%)	[17]

**Table 2:** Kruskal–Wallis test results for the effect of pretreatments on the volatile compounds of orange, mandarin, clementine hydrolates.

Pretreatment	Chi-Squared	*p*-Value
HD	5.364	0.068
HDW–RE	7.489	0.023
HDW–REP	3.918	0.141
HDW–REC	4.548	0.102
HDW–REX	4.122	0.127
HDW–REPCX	2.536	0.281
HDB–RE	5.536	0.062
HDB–REP	1.539	0.463
HDB–REC	11.104	0.003
HDB–REX	9.404	0.009
HDB–REPCX	8.083	0.017

The significance was determined at a *p*-value threshold of 0.05. Effect of different hydrodistillation pretreatments on the yield of orange, mandarin, and clementine hydrolate. HD—hydrodistillation without pretreatment (no-pretreatment control); HDW—hydrodistillation with water (no-enzyme control); HDB—hydrodistillation with buffer (no-enzyme control); RE—reflux extraction without enzyme; REP—reflux extraction with pretreatment assisted with enzyme pectinase; REC—reflux extraction with pretreatment assisted with enzyme cellulase; REX—reflux extraction with pretreatment assisted with enzyme xylanase; REPCX—reflux extraction with pretreatment assisted with mixture of enzymes (pectinase/cellulose/xylanase).

## Data Availability

The original contributions presented in this study are included in the article/Supplementary Material. Further inquiries can be directed to the corresponding authors.

## Supplementary Materials

The following supporting information can be downloaded at: https://www.mdpi.com/article/10.3390/molecules31071118/s1, Table S1. Volatile composition in orange (*Citrus sinensis*) peel hydrolate determined by HS–SPME GC–MS analysis; Table S2. Volatile composition in mandarin (*Citrus reticulata*) peel hydrolate determined by HS–SPME GC–MS analysis; Table S3. Volatile composition in clementine (*Citrus clementine*) peel hydrolate determined by HS–SPME GC–MS analysis.

## Abbreviations

The following abbreviations are used in this manuscript:

HD	hydrodistillation without pretreatment (no-pretreatment control)
HDW–RE	hydrodistillation with reflux extraction pretreatment in water (no-enzyme control)
HDW–REP	hydrodistillation with reflux extraction pretreatment assisted by pectinase enzymes in purified water
HDW–REC	hydrodistillation with reflux extraction pretreatment assisted by cellulase enzymes in purified water
HDW–REX	hydrodistillation with reflux extraction pretreatment assisted by xylanase enzymes in purified water
HDW–REPCX	hydrodistillation with reflux extraction pretreatment assisted by pectinase/cellulase/xylanase enzymes in purified water
HDB–RE	hydrodistillation with reflux extraction pretreatment in citrate buffer (no-enzyme control)
HDB–REP	hydrodistillation with reflux extraction pretreatment assisted by pectinase enzymes in citrate buffer
HDB–REC	hydrodistillation with reflux extraction pretreatment assisted by cellulase enzymes in citrate buffer
HDB–REX	hydrodistillation with reflux extraction pretreatment assisted by xylanase enzymes in citrate buffer
HDB–REPCX	hydrodistillation with reflux extraction pretreatment assisted by pectinase/cellulase/xylanase enzymes in citrate buffer
