# Supplementary material for: Citrus Peel Hydrolates as By-Products of Hydrodistillation: Volatile Characterisation and the Role of Enzymatic Pretreatment
Source: Molecules. 2026 Mar 28;31(7):1118. doi: 10.3390/molecules31071118 (PMC13074767; doi:10.3390/molecules31071118)
Supplement: Supplementary file 1 [file molecules-31-01118-s001.zip › molecules-4191251-supplementary.pdf]

Supplementary Material

Citrus Peel Hydrolates as By-Products of Hydrodistillation: Volatile Characterisation and the Role of Enzymatic Pretreatment

Maja Dent [1,*], Marija Penić [1], Antonela Ninčević Grassino [1], Krunoslav Aladić [2], Stela Jokić [2] and Igor Jerković [3,4,*]

1 Faculty of Food Technology and Biotechnology, University of Zagreb, Pierottijeva 6, 10000 Zagreb, Croatia; doktoricamare@gmail.com (M.P.); aninc@pbf.hr (A.N.G.)
2 Faculty of Food Technology Osijek, University of Josip Juraj Strossmayer in Osijek, Franje Kuhača 18, 31000 Osijek, Croatia; krunoslav.aladic@ptfos.hr (K.A.); sjokic@ptfos.hr (S.J.)
3 Faculty of Chemistry and Technology, University of Split, Ruđera Boškovića 35, 21000 Split, Croatia
4 Mediterranean Institute for Life Sciences, University of Split, Meštrovićevo šetalište 45, 21000 Split, Croatia
* Correspondence: maja.dent@pbf.unizg.hr (M.D.); igor@ktf-split.hr or igor.jerkovic@unist.hr (I.J.); Tel.: +385-1-4605-096 (M.D.)

Table S1. Volatile composition in orange (*Citrus sinensis*) peel hydrolate determined by HS-SPME GC-MS analysis

	Group	Compound	RI	% Total peak area										
				HD	HDW-RE	HDW-REP	HDW-REC	HDW-REX	HDW-REPCX	HDB-RE	HDB-REP	HDB-REC	HDB-REX	HDB-REPCX
Monoterpenes	Monoterpene hydrocarbons	*β*-Myrcene	993	-	0.02	-	0.07	-	-	0.04	-	0.05	-	0.06
		α-Phellandrene	1008	0.65	0.27	0.28	0.60	0.21	0.22	0.15	0.10	0.25	0.35	-
		α-Terpinene	1022	0.09	0.04	-	0.09	-	-	0.02	-	-	-	0.05
		ρ-Cymene	1030	0.48	0.16	0.22	0.39	0.22	0.17	0.13	0.35	0.26	0.42	-
		Limonene	1034	2.21	0.28	0.62	1.50	-	0.23	0.08	0.41	0.13	0.53	-
		1,8-Cineole	1037	-	0.09	-	-	0.47	-	-	-	0.04	0.53	-
	Oxygenated monoterpenes	6-Methylhept-5-en-2-one	989	-	0.04	-	-	-	-	0.04	-	-	-	-
		trans-Linalool oxide	1077	0.69	0.37	0.76	0.57	1.62	0.47	0.28	0.57	0.33	1.17	0.29
		cis-Linalool oxide	1092	0.74	0.18	0.73	0.55	2.13	0.42	0.24	0.60	0.29	1.44	0.14
		Linalool	1101	10.31	9.04	3.89	8.81	0.61	5.47	5.10	3.87	5.75	5.83	11.15
		cis-*p*-Ment-2-en-1-ol	1125	1.11	0.89	0.71	0.89	0.57	0.56	0.80	1.15	1.08	1.40	0.79
		trans-*p*-Ment-2-en-1-ol	1127	-	-	-	-	-	0.39	-	-	-	-	-
		cis-*p*-Menth-2,8-dienol	1139	0.88	0.48	0.44	0.78	0.64	0.50	0.70	1.12	0.99	1.26	0.54
		trans-Limonene oxide	1142	0.30	0.31	0.26	0.30	0.05	0.12	0.19	0.25	0.21	0.18	-
		β-Terpineol	1150	1.72	3.01	6.59	2.18	1.25	1.26	0.72	1.27	0.75	3.01	-
		Isopulegol	1161	-	0.11	-	-	-	0.07	-	-	-	-	-
		4-Terpineol	1181	6.72	8.60	4.19	4.84	1.02	3.59	4.95	4.19	5.35	5.45	7.45
		p-Cymen-8-ol	1185	0.53	-	0.54	0.42	0.38	-	-	-	-	-	0.25
		trans-*p*-Menth-1(7),8-dien-2-ol	1190	0.32	0.50	0.17	0.28	0.10	0.83	0.86	0.75	0.59	0.41	-
		α-Terpineol	1193	32.49	32.73	37.52	32.50	17.55	31.45	30.27	30.10	31.36	34.42	29.94
		Limonen-8,9-oxide*	1197	2.86	1.55	4.19	0.51	1.94	2.60	0.96	1.89	1.18	0.50	0.94
		trans-isopiperitenol	1202	2.18	1.36	1.98	1.75	1.30	1.53	1.91	2.48	2.10	2.55	1.45
		Barbenone	1211	1.20	0.39	0.86	0.77	1.52	0.98	1.06	1.46	0.93	1.12	-
		trans-Carveol	1222	3.40	3.79	1.00	3.20	2.25	5.16	5.84	4.58	5.39	2.98	4.10
		cis-Carveol	1234	1.17	1.52	0.60	1.52	0.65	2.16	1.94	2.17	2.22	1.12	6.07
		Nerol	1232	2.18	3.70	2.27	4.29	0.52	4.41	4.50	3.00	4.73	0.90	-
		β-Citronellol	1232	-	-	-	-	-	-	-	-	-	-	-
		(Z)-Citral	1244	0.52	2.47	0.15	1.11	0.10	0.95	2.81	0.65	1.08	0.12	3.66
		Carvone	1248	0.25	0.69	0.05	0.38	0.10	0.62	0.87	0.39	0.56	0.51	0.98
		Geraniol	1258	1.06	2.44	0.76	2.24	0.05	3.52	4.20	2.03	3.24	0.41	3.37
		(*E*)-Citral	1273	-	3.19	-	2.78	-	-	4.94	-	-	-	4.74
		cis-Isopiperitenone	1274	1.92	1.04	1.09	-	0.98	2.41	-	2.74	2.93	0.63	-
		trans-Carvone oxide	1280	-	-	-	-	-	-	0.02	-	0.12	-	-
		Thymol	1290	-	-	-	-	-	-	-	-	-	0.57	0.30
		p-Mentha-1,8(10)-dien-9-ol	1292	3.31	3.55	2.09	4.30	1.41	5.23	8.55	4.37	5.27	1.26	4.83
		Perilla alcohol	1299	0.39	0.51	0.26	0.53	0.05	0.74	0.46	0.57	0.74	0.05	0.57
		Carvacrol	1305	-	-	-	-	-	-	0.48	-	-	-	0.25
		Limonen-1,2-diol	1343	-	0.42	-	-	-	-	-	-	0.24	0.05	-
		Piperitenone	1344	-	-	-	-	-	0.48	-	-	-	-	-
	Total monoterpenes			79.68	83.74	72.22	78.15	37.69	76.54	83.11	71.06	78.16	69.17	81.92
Sesquiterpenes	Oxygenated sesquiterpenes	Viridiflorol	1592	-	0.18	-	-	-	-	0.01	-	-	-	-
		α-Copaene-8-ol	1630	-	-	-	-	-	0.18	0.12	0.32	0.06	0.20	0.03
		γ-Eudesmol	1633	-	-	-	-	-	0.22	0.09	-	0.20	-	0.04
		α-Cadinol	1644	-	-	-	0.07	-	0.16	0.26	0.21	0.10	0.13	0.04
		t-Cadinol	1656	-	0.13	0.06	-	-	0.17	0.12	-	0.20	-	0.03
	Total sesquiterpenes			-	0.31	0.06	0.07	-	0.73	0.60	0.53	0.56	0.33	0.14
Alcohols		2-Methylbut-3-en-2-ol	<800	1.86	0.37	2.60	1.83	11.21	0.93	0.37	1.28	0.72	2.84	0.28
		3-Methylbut-3-en-1-ol	781	3.74	0.88	5.01	4.65	15.32	2.24	0.72	4.08	1.31	2.56	0.50
		(Z)-Hex-3-en-1-ol	861	2.36	1.60	2.38	2.08	3.03	2.82	0.50	3.61	3.88	1.07	-
		Hexan-1-ol	872	0.96	0.85	0.77	0.50	1.17	1.94	-	1.01	1.77	0.12	-
		Heptan-1-ol	972	0.10	0.21	0.07	0.10	0.05	0.10	0.16	0.09	0.23	-	0.25
		Octan-1-ol	1074	0.60	1.27	0.24	0.70	0.20	0.55	1.05	0.30	0.63	0.12	2.06
		Nonan-1-ol	1175	-	-	-	0.12	-	-	-	-	-	-	0.12
	Total alcohols			9.62	5.18	11.07	9.98	30.98	8.58	2.80	10.37	8.54	6.71	3.21
Aldehydes		Hexanal	805	0.18	0.17	-	-	-	0.15	0.12	0.44	0.20	-	0.19
		Furfural	841	0.15	0.92	4.86	0.15	1.76	4.00	0.33	3.39	2.44	0.33	0.01
		(E)-Hex-2-enal	858	-	1.09	-	-	-	-	3.18	-	-	-	3.81
		Heptanal	903	-	-	-	-	-	-	-	-	0.04	-	-
		Benzaldehyde	968	-	0.05	-	-	-	0.06	0.11	0.05	0.07	-	-
		Octanal	1005	0.08	0.37	-	0.28	-	0.08	0.10	0.04	0.05	-	0.13
		Nonanal	1105	-	-	-	-	-	-	-	-	0.15	-	-
		Dodecanal	1410	-	-	-	-	-	-	-	-	0.13	-	-
	Total aldehydes			0.41	2.60	4.86	0.43	1.76	4.29	3.84	3.92	3.08	0.33	4.14

Others		Propan-2-one	<800	2.64	1.18	4.38	5.21	14.36	1.61	-	3.69	1.99	11.12	0.05
	Total others			2.64	1.18	4.38	5.21	14.36	1.61	-	3.69	1.99	11.12	0.05
	Total:			92.35	93.01	92.59	93.84	84.79	91.75	90.35	89.57	92.33	87.66	89.46

RI - Retention index on HP-5MS column; Effect of different hydrodistillation pretretments on the yield of orange hydrolate. HD - hydrodistillation without pretreatment (no-pretreatment control); HDW – hydrodistillation with water (no-enzyme control); HDB - hydrodistillation with buffer (no-enzyme control); RE – reflux extraction without enzyme; REP - reflux extraction with pretreatment assisted with enzyme pectinase; REC - reflux extraction with pretreatment assisted with enzyme cellulase; REX - reflux extraction with pretreatment assisted with enzyme xylanase; REPCX - reflux extraction with pretreatment assisted with mixture of enzymes (pectinase/cellulase/xylanase).

Table S2. Volatile composition in mandarin (*Citrus reticulata*) peel hydrolate determined by HS-SPME GC-MS analysis

	**Group**	**Compound**	**RI**	**% Total peak area**										
				HD	HDW-RE	HDW-REP	HDW-REC	HDW-REX	HDW-REPCX	HDB-RE	HDB-REP	HDB-REC	HDB-REX	HDB-REPCX
Monoterpenes	Monoterpene hydrocarbons	*α*-Terpinene	1022	-	-	-	0.08	-	-	-	-	-	-	-
		ρ-Cymene	1030	1.14	0.84	0.52	0.56	-	-	-	0.10	0.11	0.17	0.14
		Limonene	1034	0.37	0.35	0.26	0.67	0.26	0.12	0.42	0.18	0.45	0.73	0.15
	Oxygenated monoterpenes	*trans*-Linalool oxide	1077	0.74	0.64	0.50	0.35	2.32	4.30	2.29	1.02	0.61	1.28	1.49
		cis-Linalool oxide	1092	1.61	1.03	1.05	0.21	1.84	3.13	1.99	0.75	0.44	0.88	1.01
		Linalool	1101	0.62	2.06	0.49	2.44	0.43	0.29	0.69	1.28	1.94	0.16	0.13
		cis-p-Ment-2-en-1-ol	1125	1.21	1.95	0.61	1.27	0.39	0.39	0.57	0.35	0.34	0.28	0.47
		trans-p-Ment-2-en-1-ol	1127	-	-	-	-	-	0.39	-	-	-	-	-
		cis-p-Menth-2,8-dienol	1139	1.63	2.04	1.06	1.24	0.50	0.57	0.70	0.36	0.31	0.33	0.51
		trans-Limonene oxide	1142	0.26	-	-	1.24	-	-	-	-	-	-	-
		β-Terpineol	1150	0.35	0.95	0.51	0.92	0.25	0.38	0.22	0.22	0.27	0.15	0.24
		4-Terpineol	1181	1.47	3.08	1.01	3.65	0.62	0.65	1.28	1.22	1.80	1.02	1.74
		p-Cymen-8-ol	1185	-	-	1.35	1.34	1.06	-	-	-	-	-	-
		trans-p-Menth-1(7),8-dien-2-ol	1190	1.17	1.30	-	-	-	1.49	1.40	0.67	0.74	0.56	0.81
		α-Terpineol	1193	27.88	27.95	26.97	35.51	16.27	27.12	37.29	61.57	69.14	67.56	60.94
		trans-isopiperitenol	1202	2.95	2.75	2.36	2.25	0.49	1.46	1.56	1.47	1.52	1.63	1.63
		Barbenone	1211	1.74	1.10	1.08	0.58	0.61	0.72	0.82	0.40	0.48	0.55	0.46
		trans-Carveol	1222	4.39	5.95	4.65	4.79	3.00	3.40	3.04	1.63	2.44	1.64	2.25
		cis-Carveol	1234	0.95	1.43	1.05	1.43	0.64	0.88	0.79	0.47	0.76	0.49	0.42
		Nerol	1232	0.78	0.99	0.78	1.91	0.36	0.63	0.80	-	0.40	0.25	-
		(Z)-Citral	1244	-	0.06	-	-	-	-	-	-	-	-	-
		Carvone	1248	-	2.44	-	0.38	0.41	-	-	-	0.79	0.10	0.03
		Geraniol	1258	0.45	0.51	0.59	2.01	0.29	0.53	0.56	0.14	0.10	0.12	0.11
		cis-Isopiperitenone	1274	0.28	0.58	0.35	0.33	0.41	0.52	1.66	0.67	0.74	0.62	1.20
		trans-Carvone oxide	1280	-	-	-	-	-	-	0.02	-	0.12	-	-
		Thymol	1290	-	-	-	-	-	-	-	-	-	0.57	0.30
		p-Mentha-1,8(10)-dien-9-ol	1292	5.50	3.69	5.47	5.37	4.12	4.85	5.01	1.24	0.81	1.22	1.13
		Perilla alcohol	1299	3.80	2.05	3.97	5.41	5.25	8.03	7.23	2.18	1.31	2.86	2.81
		Carvacrol	1305	2.36	1.30	1.88	2.91	2.91	2.05	3.87	1.94	0.88	1.82	0.88
		Limonen-1,2-diol	1343	-	0.42	-	-	-	-	-	-	0.24	0.05	-
	Total monoterpenes			61.65	65.46	56.51	76.85	42.43	61.90	72.21	77.86	86.74	85.04	78.85
Sesquiterpenes	Oxygenated sesquiterpenes	Viridiflorol	1592	-	-	-	0.19	-	0.25	0.05	0.11	0.16	0.13	0.05
		α-Copaene-8-ol	1630	-	-	-	-	-	0.18	0.12	0.32	0.06	0.20	0.03
		γ-Eudesmol	1633	-	-	0.29	0.22	0.57	0.55	0.58	0.42	0.10	0.25	0.09
		α-Cadinol	1644	-	-	-	0.07	-	0.16	0.26	0.21	0.10	0.13	0.04
		t-Cadinol	1656	0.45	0.18	0.96	0.76	3.62	2.59	2.22	2.09	0.36	1.05	0.35
	Total sesquiterpenes			0.45	0.18	1.25	1.24	4.19	3.73	3.23	3.15	0.78	1.76	0.56
Alcohols		2-Methylbut-3-en-2-ol	<800	3.44	1.45	5.80	0.46	12.20	3.07	1.32	0.94	0.62	0.93	0.78
		3-Methylbut-3-en-1-ol	781	6.71	3.00	9.46	1.01	9.65	5.53	2.32	2.17	1.10	0.82	1.12
		(Z)-Hex-3-en-1-ol	861	5.94	8.39	3.93	-	2.79	2.53	1.99	1.10	0.66	0.77	2.36
		Hexan-1-ol	872	1.70	3.24	1.64	6.75	2.30	3.99	2.40	1.14	0.56	0.50	1.54
		Heptan-1-ol	972	-	0.14	-	0.21	-	-	-	-	0.04	-	-
		Octan-1-ol	1074	-	0.06	-	0.48	-	-	0.15	0.23	0.21	-	-
		Nonan-1-ol	1175	-	-	-	-	-	-	-	-	0.16	-	-
	Total alcohols			17.79	16.28	20.83	8.91	26.94	15.12	8.18	5.58	3.35	3.02	5.80
		Hexanal	805	-	-	-	0.17	-	-	-	-	0.21	-	-

Alde-hydes		Furfural	841	0.21	0.18	1.94	0.28	3.75	3.52	1.68	1.51	1.20	0.53	3.50
		(E)-Hex-2-enal	858	-	-	-	3.91	-	-	-	-	-	-	-
		Heptanal	903	-	-	-	-	-	-	-	-	0.04	-	-
		Benzaldehyde	968	-	-	-	-	-	-	-	-	-	-	0.10
		Octanal	1005	-	0.04	-	-	-	-	-	-	0.28	-	-
		Nonanal	1105	-	-	-	-	-	-	-	-	0.15	-	-
		Dodecanal	1410	-	-	-	-	-	-	-	-	0.13	-	-
	Total aldehydes			0.21	0.22	1.94	4.36	3.75	3.52	1.68	1.51	2.01	0.53	3.60
Others		Propan-2-one	<800	7.30	4.42	6.73	0.79	9.66	4.15	2.50	0.77	1.02	0.79	0.53
	Total others			7.30	4.42	6.73	0.79	9.66	4.15	2.50	0.77	1.02	0.79	0.53
	Total:			87.40	86.56	87.26	92.15	86.97	88.42	87.80	88.87	93.90	91.14	89.34

RI - Retention index on HP-5MS column; Effect of different hydrodistillation pretretments on the yield of mandarin hydrolate. HD - hydrodistillation without pretreatment (no-pretreatment control); HDW – hydrodistillation with water (no-enzyme control); HDB - hydrodistillation with buffer (no-enzyme control); RE – reflux extraction without enzyme; REP - reflux extraction with pretreatment assisted with enzyme pectinase; REC - reflux extraction with pretreatment assisted with enzyme cellulase; REX - reflux extraction with pretreatment assisted with enzyme xylanase; REPCX - reflux extraction with pretreatment assisted with mixture of enzymes (pectinase/cellulase/xylanase).

Table S3. Volatile composition in clementine (*Citrus clementine*) peel hydrolate determined by HS-SPME GC-MS analysis

	**Group**	**Compound**	**RI**	**% Total peak area**										
				HD	HDW-RE	HDW-REP	HDW-REC	HDW-REX	HDW-REPCX	HDB-RE	HDB-REP	HDB-REC	HDB-REX	HDB-REPCX
Monoter-penes	Monoterpene hy-drocarbons	*β*-Myrcene	993	0.04	-	-	-	-	-	-	-	-	-	-
		α-Phellandrene	1008	0.38	-	0.21	-	-	-	-	-	-	-	-
		α-Terpinene	1022	0.07	-	-	-	-	-	-	-	-	-	-
		ρ-Cymene	1030	-	-	0.30	-	0.54	0.46	0.78	0.45	0.70	0.63	0.85
		Limonene	1034	0.25	0.27	-	-	2.30	1.06	0.31	0.07	0.45	1.45	0.64
	Oxygenated mon-oterpenes	6-Methylhept-5-en-2-one	989	0.05	-	-	-	-	-	-	-	-	-	-
		trans-Linalool ox-ide	1077	0.50	2.36	2.14	2.90	1.64	1.64	1.21	0.49	1.80	1.45	3.22
		cis-Linalool oxide	1092	0.44	4.11	3.12	4.01	2.12	1.92	1.76	0.67	3.01	2.36	4.79
		Linalool	1101	16.45	2.41	3.54	1.20	2.79	2.62	4.40	6.90	6.67	2.68	1.74
		cis-*p*-Ment-2-en-1-ol	1125	1.78	1.33	1.18	1.19	1.56	1.83	1.87	2.06	2.08	1.85	1.94
		cis-*p*-Menth-2,8-dienol	1139	1.28	0.93	0.96	0.70	1.10	1.27	1.57	1.81	1.58	1.46	1.44
		trans-Limonene ox-ide	1142	0.28	-	0.15	-	0.13	0.13	0.21	0.22	0.20	0.17	-
		β-Terpineol	1150	1.11	-	0.75	-	1.04	1.15	0.70	0.90	0.88	0.75	0.36
		Isopulegol	1161	0.30	-	-	-	-	-	-	0.05	-	-	-
		4-Terpineol	1181	7.65	0.87	0.87	0.65	2.29	2.86	3.59	5.26	2.76	2.23	1.23
		p-Cymen-8-ol	1185	-	0.30	0.37	0.52	0.61	0.77	0.73	1.13	-	0.62	0.50
		trans-*p*-Menth-1(7),8-dien-2-ol	1190	0.43	-	-	-	-	-	0.56	1.01	0.38	0.62	0.27
		α-Terpineol	1193	25.92	16.40	25.11	11.09	28.86	28.39	28.21	15.17	23.27	23.36	20.26
		Limonen-8,9-ox-ide*	1197	0.39	-	-	1.65	-	1.75	2.79	0.38	-	-	-
		trans-isopiperitenol	1202	2.28	0.89	2.00	1.26	2.54	2.46	0.90	2.39	2.49	2.56	2.47
		Barbenone	1211	-	-	1.58	-	1.43	1.22	1.46	0.68	1.77	1.63	2.42
		trans-Carveol	1222	4.73	3.35	3.08	2.56	5.47	6.68	7.36	13.63	4.94	5.85	4.42
		cis-Carveol	1234	1.77	1.35	1.02	0.69	2.23	2.08	2.58	4.25	1.69	2.91	1.32
		Nerol	1232	2.88	1.28	1.40	0.75	1.80	1.49	2.13	1.76	1.53	1.87	1.26
		(Z)-Citral	1244	1.00	-	-	-	-	-	-	-	-	-	-
		Carvone	1248	1.81	-	-	-	0.39	1.11	1.20	8.00	0.46	-	0.22
		Geraniol	1258	1.73	-	-	-	0.43	0.34	0.35	0.41	0.20	0.36	-
		cis-Isopiperitenone	1274	4.93	8.88	6.57	6.27	7.45	6.46	8.25	4.11	8.04	9.65	9.20
		Perilla aldehyde	1277	1.10	-	-	-	-	-	-	-	-	-	-
		trans-Carvone ox-ide	1280	-	-	-	-	-	-	-	0.53	0.04	0.40	-
		p-Mentha-1,8(10)-dien-9-ol	1292	3.17	4.28	2.73	1.46	4.76	3.83	5.27	2.67	4.31	6.02	-
		Perilla alcohol	1299	0.77	1.02	0.89	0.47	1.10	1.22	1.12	1.55	0.96	1.34	3.90
		Carvacrol	1305	0.22	-	-	-	-	-	-	-	-	-	-
		Limonen-1,2-diol	1343	-	-	-	-	-	-	-	4.50	0.28	0.17	0.04
		Piperitenone	1344	0.13	-	-	-	0.01	-	0.45	-	-	-	-
		(*Z*)-*p*-Mentha-1,8-dien-2-hydroper-oxide	1367	-	-	-	-	-	-	-	1.31	-	-	-
		(*E*)-*p*-Mentha-6,8-dien-2-hydroperoxide	1381	-	-	-	-	-	-	-	0.52	-	-	-

	Total monoterpenes			83.84	50.03	57.97	37.37	72.59	72.74	79.76	82.88	70.49	72.39	62.49
Sesquiterpenes														
	Oxygenated sesquiterpenes	Viridiflorol	1592	-	0.56	0.38	0.30	0.46	0.57	0.23	0.10	-	0.45	0.19
		α-Copaene-8-ol	1630	-	-	-	-	-	-	-	-	-	0.20	-
		γ-Eudesmol	1633	0.04	-	-	-	0.15	0.33	0.13	0.06	-	0.40	-
		α-Cadinol	1644	-	-	-	-	-	-	-	-	-	0.18	-
		t-Cadinol	1656	-	-	-	0.24	0.13	0.30	0.12	0.07	0.15	1.11	0.24
	Total sesquiterpenes			0.04	0.56	0.38	0.54	0.74	1.20	0.48	0.23	0.15	2.34	0.43
Alcohols		2-Methylbut-3-en-2-ol	<800	0.29	14.02	5.95	10.42	2.24	1.77	1.39	1.00	2.27	2.12	4.72
		3-Methylbut-3-en-1-ol	781	0.62	7.65	10.68	16.75	4.93	4.61	3.02	1.15	4.30	3.63	7.94
		(Z)-Hex-3-en-1-ol	861	-	1.18	2.27	3.08	2.09	2.74	1.67	-	3.31	2.21	3.70
		Hexan-1-ol	872	0.69	-	-	-	-	1.18	0.43	0.64	1.08	0.47	1.57
		Heptan-1-ol	972	0.40	-	-	-	-	0.06	0.07	0.13	-	-	-
		Octan-1-ol	1074	1.60	-	-	-	-	0.06	0.10	0.22	0.11	-	-
	Total alcohols			3.60	22.85	18.90	30.25	9.26	10.42	6.68	3.14	11.07	8.43	17.93
Aldehydes		Hexanal	805	0.21	-	-	-	-	0.26	-	0.19	-	0.49	-
		Furfural	841	0.12	0.01	7.56	10.73	4.28	3.93	1.73	0.94	2.92	2.19	0.32
		(E)-Hex-2-enal	858	3.92	-	-	-	-	-	-	1.81	-	-	-
		Heptanal	903	-	-	-	-	-	-	-	0.05	-	-	-
		Benzaldehyde	968	0.12	-	-	-	-	0.05	-	0.03	-	-	-
		Octanal	1005	0.17	-	-	-	-	-	0.22	0.31	0.15	-	-
	Total aldehydes			4.54	0.01	7.56	10.73	4.28	4.24	1.95	3.33	3.07	2.68	0.32
Others		Propan-2-one	<800	0.02	13.68	6.10	8.00	3.72	2.79	3.95	1.78	4.12	5.38	6.63
	Total others			0.02	13.68	6.10	8.00	3.72	2.79	3.95	1.78	4.12	5.38	6.63
	Total:			92.04	87.13	90.91	86.89	90.59	91.39	92.82	91.36	88.90	91.22	87.80

RI - Retention index on HP-5MS column; Effect of different hydrodistillation pretretments on the yield of clementine hydrolate. HD - hydrodistillation without pretreatment (no-pretreatment control); HDW – hydrodistillation with water (no-enzyme control); HDB - hydrodistillation with buffer (no-enzyme control); RE – reflux extraction without enzyme; REP - reflux extraction with pretreatment assisted with enzyme pectinase; REC - reflux extraction with pretreatment assisted with enzyme cellulase; REX - reflux extraction with pretreatment assisted with enzyme xylanase; REPCX - reflux extraction with pretreatment assisted with mixture of enzymes (pectinase/cellulase/xylanase).
